# Modeling Nitrogen Fate and Water and Nitrogen Use Efficiencies under Different Greenhouse Vegetable Production Systems Using the WHCNS-Veg Model

**DOI:** 10.3390/plants13101384

**Published:** 2024-05-16

**Authors:** Hongyuan Zhang, William D. Batchelor, Kelin Hu, Hui Han, Ji Li

**Affiliations:** 1 School of Agriculture, Ludong University, Yantai 264025, China; zhy20074014@163.com; 2 College of Land Science and Technology, China Agricultural University, Key Laboratory of Arable Land Conservation (North China), Ministry of Agriculture, Beijing 100193, China; 3 Biosystems Engineering Department, Auburn University, Auburn, AL 36849, USA; 4 College of Resources and Environmental Sciences, China Agricultural University, Key Laboratory of Biodiversity and Organic Farming, Beijing 100193, China

**Keywords:** greenhouse vegetable production system, gaseous N loss, nitrate leaching, water and nitrogen utilization efficiencies, WHCNS-Veg model

## Abstract

Quantitative evaluation of the effects of diverse greenhouse vegetable production systems (GVPS) on vegetable yield, soil water consumption, and nitrogen (N) fates could provide a scientific basis for identifying optimum water and fertilizer management practices for GVPS. This research was conducted from 2013 to 2015 in a greenhouse vegetable field in Quzhou County, North China. Three production systems were designed: conventional (CON), integrated (INT), and organic (ORG) systems. The WHCNS-Veg model was employed for simulating vegetable growth, water dynamics, and fates of N, as well as water and N use efficiencies (WUE and NUE) for four continuous growing seasons. The simulation results revealed that nitrate leaching and gaseous N emissions constituted the predominant N loss within GVPS, which separately accounted for 11.5–59.4% and 6.0–21.1% of the N outputs. The order of vegetable yield, N uptake, WUE, and NUE under different production systems was ORG > INT > CON, while the order of nitrate leaching and gaseous N loss was CON > INT > ORG. Compared to CON, ORG exhibited a significant increase in yield, N uptake, WUE, and NUE by 24.6%, 24.2%, 26.1%, and 89.7%, respectively, alongside notable reductions in nitrate leaching and gaseous N loss by 67.7% and 63.2%, respectively. The ORG system should be recommended to local farmers.

## 1. Introduction

Over the last three decades, China’s vegetable planting area has surged from 3.1 × 10^6^ ha^−1^ in 1980 to 2.1 × 10^7^ ha^−1^ in 2020, a seven-fold increase [1]. China is the world’s leading vegetable production country, producing nearly half of the world’s vegetables using 41% of the global vegetable production area [2]. At the same time, China’s vegetable production system is intensive and characterized by large amounts of water and fertilizer inputs, which are accompanied by a large loss of nitrogen (N). Annual N leaching can reach 79.1 kg N ha^−1^ [3], and the annual N_2_O emission can reach 3.91 kg N ha^−1^ [4]. These losses stem from excessive fertilizer use within China’s vegetable production system. Research indicates that the average N application rate in a single season in a typical vegetable production system is 423 kg N ha^−1^, much greater than that within the field crop production system (171–249 kg N ha^−1^) [5,6]. Vegetable roots are mainly distributed in the top 30 cm of soil, and it is difficult for the crop to utilize the deep soil residual N. Excessive irrigation leads to nitrate leaching, heightening the risk of groundwater contamination.

In recent years, environmental pollution due to N leaching in vegetable growing areas has become a public concern. Qasim et al. (2021) synthesized findings from 75 studies focused on greenhouse vegetable systems and found N leaching from greenhouse vegetable production contributes 1.66 Tg N yr^−1^ to groundwater pollution [7]. The nitrate concentration surpassed the drinking water standard (10 mg N L^−1^) in around 26% of the monitored drinking wells. In their investigation of intensive greenhouse vegetable fields in the North China Plain (NCP), Zhang et al. (2010) observed nitrate levels in shallow groundwater ranging from 25.3 to 279.6 mg N L^−1^, averaging 121.6 mg N L^−1^. They reported that 87% of water samples exceeded the European drinking water quality standard (11.3 mg N L^−1^) [8]. Pang et al. (2013) noted that groundwater pollution in vegetable cultivation areas of the NCP was more severe compared to the surrounding farmland [9]. Nitrate content in groundwater was as high as 258.0 mg N L^−1^, with an average of 86.8 mg N L^−1^, which posed a great threat to human health. In addition, N_2_O emissions, as well as other greenhouse gases (GHG), increase due to overuse of N fertilizer [4], thus increasing global warming. 

These traditional vegetable production systems exhibit substantial water and fertilizer usage, accompanied by low efficiencies in water and nitrogen utilization (WUE and NUE) and large amounts of nitrate leaching [10,11]. Numerous studies have shown that optimizing N rates and adjusting water irrigation strategies can effectively reduce nitrate leaching in greenhouse vegetable fields [3,10]. In contrast to conventional production systems, organic production systems do not use chemical fertilizers, leading to decreased N loss and energy consumption. These systems also protect the environment and contribute to the sustainable development of agriculture [12,13]. Some studies have shown that vegetable yields in organic production systems are generally lower than in conventional production systems [14], but organic production systems significantly reduce N leaching losses [12,13] and gaseous N loss [15], thereby reducing the negative impact on the environment. 

The transformation process of soil N is very complex, including nitrification, denitrification, NH_3_ volatilization, leaching, and other processes. The traditional experimental method to evaluate these processes is expensive and time consuming, and difficult to monitor continuously throughout the entire season. Therefore, soil–crop models have gained widespread usage in recent years to evaluate optimum N management practices [16,17,18]. Expert-N [19] and N_ABLE [16] were two of the earliest models applied to water and fertilizer management for vegetables. Some vegetable models have been developed on the foundation of the N_ABLE model, such as WELL_N [20], NPK model [21], SMCR_N [22], and EU-Rotate_N [18]. The EU-Rotate_N model is extensively utilized for simulating vegetable growth, soil water movement, and nitrogen (N) transport across various water and N management strategies [10,23,24]. However, the EU-Rotate_N model frequently overestimates N leaching due to its utilization of a simplistic water balance method for simulating soil water movement [23]. Liang et al. (2018) incorporated the vegetable growth component of EU-Rotate_N into the WHCNS model to overcome limitations in simulating soil water balance for greenhouse vegetable fields [25]. However, more studies are needed to evaluate soil N leaching, gaseous N loss, and WUE and NUE under different vegetable production systems.

In recent years, organic fertilizer has been widely promoted in the Chinese vegetable system. Studies have shown that substituting organic fertilizers for chemical fertilizers can increase vegetable yield and decrease N_2_O emission, NH_3_ volatilization, and N leaching [26,27]. The sustained use of organic fertilizers enhanced soil quality by improving water-holding capacity, soil hydraulic conductivity, and structure. Additionally, it augmented soil organic carbon and nutrient levels while minimizing nitrogen loss and enhancing nitrogen use efficiency (NUE) [28,29,30]. There is no report on evaluating the effects of changing soil physical properties and nutrient levels caused by long-term application of organic manure on vegetable yield, N fates, and NUE.

Therefore, the aims of this study were to (1) verify the applicability of the WHCNS-Veg model to simulate vegetable growth and N fates under different vegetable production systems; (2) compare vegetable yields, crop N uptake, soil N leaching, gaseous N loss, WUE, and NUE under different production systems; and (3) determine the optimal system among the three production systems. This study was conducted within a long-term vegetable greenhouse experiment initiated in 2002, encompassing three distinct production systems: conventional (CON), integrated (INT), and organic (ORG).

## 2. Materials and Methods

### 2.1. Study Area

The study was carried out at the Experimental Station of China Agricultural University, located in the northern region of Quzhou County, Hebei Province, China (36°52′ N, 115°01′ E, elev 35–37 m). Since March 2002, a long-term experiment on vegetable growth has been ongoing in an area characterized by a continental monsoonal climate. This region benefits from abundant climate resources, including light, heat, and water, albeit with notable monsoonal influences. Spring and winter seasons tend to be characterized by dry and cold weather, while summers are warm and rainy. The average annual temperature is 13.2 ℃, with an average annual rainfall of 604 mm, the majority of which (70%) typically falls between July and September. Additionally, the average annual evaporation rate is 1841 mm. The soil in this area is classified as Aquic Cambisol, characterized by a silty loam texture, with sand content ranging from 18 to 20%, silt content from 60 to 68%, and clay content from 14 to 20%.

### 2.2. Experiment Design

To adhere to organic agriculture regulations and minimize the risk of cross-contamination in greenhouse production, the study was conducted across three adjacent greenhouses representing intensified conventional (CON), integrated (INT), and organic (ORG) farming systems. Each system consisted of three replicates, with each replicate covering an area of 120 m^2^ within separate semi-round arch greenhouses measuring 52 m in length and 7 m in width. A 3.0 m border zone was maintained at each end of the greenhouse, with a 1.0 m buffer zone between replicates to prevent any potential interference. Consistent practices of vegetable rotation, irrigation, and tillage have been implemented across all three systems since 2002. The experiment period spanned from 2013 to 2015, encompassing two growing seasons annually: the autumn–winter (AW) season from early October to early February and the spring–summer (SS) season from late February to late September.

During the AW seasons of 2013 and 2014, cauliflower (*Brassica oleracea* L. var. *botrytis* L.) and celery (*Apium graveolens* L.) were transplanted into the three systems. In addition, eggplant (*Solanum melongena* L.) was transplanted during the SS seasons of 2014 and 2015. The CON system adhered to conventional greenhouse vegetable production practices prevalent in the local area. This involved the application of chemical fertilizers, including urea, superphosphate, and potassium sulfate (N:P_2_O_5_:K_2_O, 17:17:17), solid compost derived from chicken- and cow-manure (containing 1.2% nitrogen and 13.2% carbon), the ratio of chemical fertilizers and compost is 7:3, and appropriate pesticides for plant protection. The ORG system adhered to the guidelines and principles outlined by the International Federation of Organic Agriculture Movements (IFOAM), employing 100% compost for fertilization. Additionally, it employed biological fungicides (*Bacillus subtilis*, matrine, Bamboo Vinegar, etc.) and physical methods, including the use of insect nets, yellow board trapping, and artificial insect catching, for plant protection. In contrast, the INT system incorporated a blend of 50% chemical fertilizers and 50% compost, employing biological methods for overall plant protection alongside the use of low-toxic chemical pesticides (Emamectin benzoate salt, pyrethrum, etc.). In addition, we applied the same type of fertilizer (chemical and organic) for different crops and different production systems.

Table 1 shows the types and rates of N fertilizer input for each season under different production systems. The overall fertilization rate was adjusted based on the specific growth requirements of each vegetable season, aligning with customary practices among local farmers. While the N application rates were comparable across the CON, INT, and ORG systems, slight variations occurred due to differences in compost moisture content per season. As a result, the N input in the ORG system slightly exceeded those of the CON and INT systems. During the AW season, both chemical fertilizer and compost were applied as basal fertilizer onto the surface soil and subsequently incorporated into the top 20 cm depth through plowing before transplanting across all three systems. During the SS season, only a portion of chemical fertilizer was applied as topdressing with flood irrigation. Topdressing applications were scheduled on specific dates for the CON and INT systems: 30 April, 15 June, and 15 July in 2014. The ratio between basal fertilizer and topdressing was 1.7:1 for the CON system and 2.8:1 for the INT system. In 2015, a single topdressing event occurred on 29 April, with ratios of 2.8:1 for the CON system and 5.7:1 for the INT system.

Flood irrigation practices were implemented in accordance with traditional methods employed by local farmers. Throughout the cauliflower, eggplant, celery, and subsequent eggplant growing seasons, vegetables received irrigation 3, 11, 4, and 10 times, respectively. Each irrigation event applied approximately 100 mm of water.

### 2.3. Sampling and Analysis

In April 2013, a soil profile pit was excavated to a depth of 100 cm and soil samples were collected from each soil textural layer for analysis. Various basic soil physical and chemical properties were measured. Soil bulk density was measured using the cutting rings method, while soil texture was measured via the pipette method. pH levels were determined by a pH meter with a standardized soil-to-water ratio of 1:2.5. Soil organic matter content was quantified utilizing the potassium dichromate wet combustion procedure, and total nitrogen content was assessed using the Kjeldahl method. Additionally, essential soil hydraulic properties such as soil saturated water content, field capacity, wilting point, and hydraulic conductivity were precisely determined for each layer, as outlined in Table 2. Furthermore, the initial soil nutrient contents up to September 2013 are comprehensively documented in Table 3. It is noteworthy that the levels of available phosphorus and potassium in the soil were identified as being of high grade, satisfying the optimal requirements for vegetable growth.

From October 2013 to September 2015, soil samples were systematically collected at 20 cm intervals down to a depth of 100 cm using a soil auger. Sampling occurred within 3–5 days following each irrigation event. Soil water content was measured using the oven-drying method. Nitrate concentrations were analyzed in fresh soil samples extracted with 2 mol L^−1^ KCl using a continuous flow analyzer (AA3, Bran and Luebbe, Norderstedt, Germany). During the harvest period, plants were gathered from a 6 m^2^ sample area within each replication to evaluate vegetable yields. Plant samples were subsequently dried at 75 °C to measure the plant nitrogen (N) content. Additionally, an automatic weather station (RR-9100, Rainroot, Beijing, China) was installed within the CON system greenhouse to continuously monitor meteorological parameters, including temperature, relative humidity, wind speed, and solar radiation.

### 2.4. WHCNS-Veg Model

For this investigation, the WHCNS-Veg model was employed to simulate various aspects such as soil water movement, soil heat and nitrogen transport, as well as vegetable growth. This model consisted of five principal modules, including soil water dynamics, soil temperature variations, soil carbon cycling, nitrogen transformation, and the developmental processes of vegetables. The model integrates the key processes, including soil evaporation, vegetable transpiration, mineralization and immobilization of soil organic N, NH_3_ volatilization, nitrification, denitrification, and agricultural practices.

The WHCNS-Veg model employed the grass-based Penman–Monteith method for computing the reference evapotranspiration [31]. The Green–Ampt model [32] and Richard’s equation are used to simulate soil water infiltration and redistribution, respectively. Root water uptake is computed by the van Genuchten model [33] incorporating a compensatory root water uptake mechanism [34]. Soil heat transport is modeled based on the HYDRUS-1D model [35]. The convection–diffusion equation is used to simulate soil mineral N transport, while soil C and N cycling modules are adapted from the DAISY model [36]. Ammonia volatilization is simulated using a method proposed by Freney et al. (1985) [37], and the value of pH is one of the main factors influencing NH_3_ volatilization. Based on the measurements of pH during the four seasons, we found the coefficient of variation for pH is only 0.03. Therefore, pH is fixed as a constant, and its fluctuation is ignored within the model. Vegetable growth, dry matter accumulation and allocation, fresh yield formation, and vegetable N uptake are derived from the EU_Rotate-N model [16,18]. A detailed model description is available in the literature [25]. The model operates on a daily time step, driven by meteorological and crop biological data. Model inputs encompass site specifics (latitude, altitude), fundamental soil physical–chemical properties, crop details (rotation, sowing, and harvest dates, density, and depth), field management practices (irrigation, fertilization, straw return, tillage, etc.), initial soil water and mineral N content, and daily meteorological data.

### 2.5. Model Evaluation Statistics

Three statistical indices, namely normalized root mean square error (*NRMSE*) [38], Nash–Sutcliffe modeling efficiency (*NSE*) [39], and Index of agreement (*d*) [40], were utilized to assess the agreement between the forecasted and actual datasets.

(1) $$NRMSE=\frac{100}{\bar{O}}\sqrt{\overset{n}{\underset{i=1}{\sum }}\frac{{\left(P_{i}-O_{i}\right)}^{2}}{n}}$$

(2) $$NSE=1-\frac{\sum_{i=1}^{n}{\left(O_{i}-P_{i}\right)}^{2}}{\sum_{i=1}^{n}{\left(O_{i}-\bar{O}\right)}^{2}}$$

(3) $$d=1-\frac{\sum_{i=1}^{n}{\left(O_{i}-P_{i}\right)}^{2}}{\sum_{i=1}^{n}{\left(|P_{i}-\bar{O}|+|O_{i}-\bar{O|}\right)}^{2}}$$

where *n* is the number of simulated–measured pairs of values compared. *P_i_* and *O_i_* are the simulated and measured values at time *i*, respectively. $\bar{O}$ is the mean of the measured value, Normalized Root Mean Square Error (*NRMSE*) quantifies the average deviation as a percentage relative to the measured mean. It categorizes agreement as “good” when *NRMSE* is below 15%, “moderate” when between 15% and 30%, and “poor” when exceeding 30%. Nash–Sutcliffe Efficiency (*NSE*) varies between −∞ and 1. The *d* value, ranging from 0 to 1, indicates the ratio of mean square error to potential error. The closer *NSE* (or *d*) approaches 1, the higher the model’s performance [38]. According to van Liew and Garbrecht (2003) [40], a satisfactory simulation requires *NSE* to be greater than 0.36 and *d* to exceed 0.7.

### 2.6. Data Analysis and Calculation

Data processing was performed with Microsoft Corporation’s Excel 2018 software, while graphical representations were generated using OriginLab Corporation’s Origin 2018. Statistical analysis, including analysis of variance, was conducted using International Business Machines Corporation’s SPSS 23 software. The significance of differences was evaluated through the F-test and least squares (LSD) methodology. Furthermore, the following equations were used to compute water use efficiency (WUE) and nitrogen use efficiency (NUE).

(4) WUE=Freshyield/ET

NUE = Fresh yield/(Nup + Nlea + Ngas)(5)

where ET is evapotranspiration (mm), Nup is vegetable N uptake (kg N ha^−1^), Nlea is N leaching (kg N ha^−1^), and Ngas is gaseous N loss (kg N ha^−1^).

## 3. Results

### 3.1. Model Calibration and Validation

The filed observed dataset of CON, encompassing soil water moisture, NO_3_^−^-N concentration, vegetable N uptake, and vegetable yield, was utilized for model calibration. Subsequently, the model was validated using observed datasets from both the INT and ORG systems. Soil hydraulic parameters, such as field capacity, wilting point, and saturated hydraulic conductivity, were determined from measurements and adjusted iteratively. The calibrated soil hydraulic parameters are presented in Table 2.

The N transformation parameters encompass various factors: maximum nitrification rate (*V_n_*), half saturation constant (*K_n_*), empirical proportionality factor (*K_d_*), empirical coefficient (*A_d_*), first-order kinetic constant of volatilization (*K_v_*), as well as the ratios of N_2_O produced by nitrification (*R_nit_*) and denitrification (*R_den_*) processes, respectively. These parameters were mainly derived from the simulated results of related greenhouse vegetables [25,41] and were through iterative experimentation, guided by the measured values of soil inorganic N and vegetable N uptake, to avoid redundancy (Table 4). Organic matter transformation parameters, including kinetic parameters and partition coefficients of each organic pool, were mainly derived from the studies of Hansen et al. (2012) [36] and Jensen et al. (2005) [42].

Crop growth and development parameters mainly include vegetable growth and development base temperature (*T_base_*), accumulated temperature from emergence to maturity (*T_sum_*), crop coefficient (*K_ini_*, *K_mid_*, *K_end_*), maximum root depth (*R_max_*), and empirical parameters for dry matter accumulation (*αDM*). Based on the results of previous vegetable research [25] and the measured vegetable yield, adjustments were made using a “trial and error method”. The calibrated crop parameters are presented in Table 4.

#### 3.1.1. Soil Water and Nitrate Content

**Figure 1:**
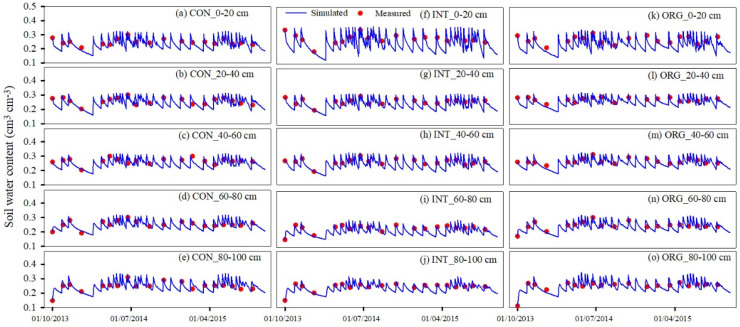
Comparison of simulated and measured soil water content under three production systems from 2013 to 2015.

Figure 1 displays the soil water moisture, both simulated and measured, across various soil layers within three distinct production systems. The simulated trends closely align with the measured values, indicating a robust agreement between simulation and observation (Figure 1). Following irrigation, there was a rapid increase in soil water moisture across all layers, which subsequently declined due to vegetable transpiration or soil evaporation, resulting in frequent water fluctuations throughout the vegetable growth period. Conversely, during the fallow period (January to February, September to October in 2014, and February to March in 2015), soil water content remained relatively stable, exhibiting no significant variation among the three production systems under identical water management conditions.

**Figure 2:**
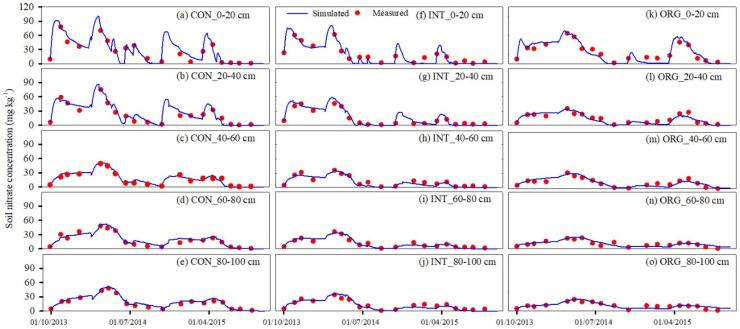
Comparison of soil nitrate concentration between simulated and measured values across three production systems during the period from 2013 to 2015.

Due to the prevalence of nitrification in upland soils of North China and consistent findings from numerous field experiments indicating significantly higher NO_3_^−^-N concentrations compared to NH_4_^+^-N concentrations, our study exclusively focuses on nitrate transport. The simulated soil nitrate concentration in each soil layer also agreed well with the measured values (Figure 2). The soil nitrate concentration in the top 40 cm soil layer fluctuated sharply, which was related to fertilizer applications. A base fertilizer was applied to all three production systems. During the plowing process, the base fertilizer was mixed into the top 20 cm soil layer. Nitrate concentration peaked after each fertilizer application. The peaks varied depending on the fertilizer type and application rate for the three production systems. The soil nitrate concentration and peaks in each soil layer in CON were the highest, and the ORG was the lowest, which was related to the larger amount of chemical fertilizer application in this system.

For model calibration, the *NRMSE* values for soil water content across soil layers ranged from 3.65% to 9.17% in the CON system (Table 4). The *NSE* values varied between 0.50 and 0.78, and the *d* values ranged between 0.88 and 0.94. For the validation systems (INT and ORG), the *NRMSE* values of the soil water content across layers ranged from 4.19% to 8.34%. The *NSE* values varied from 0.47 to 0.79, and the *d* values varied from 0.88 to 0.95. These results align well with the findings of van Liew and Garbrecht et al. (2003) [40].

#### 3.1.2. Vegetable Yield and N Uptake

**Figure 3:**
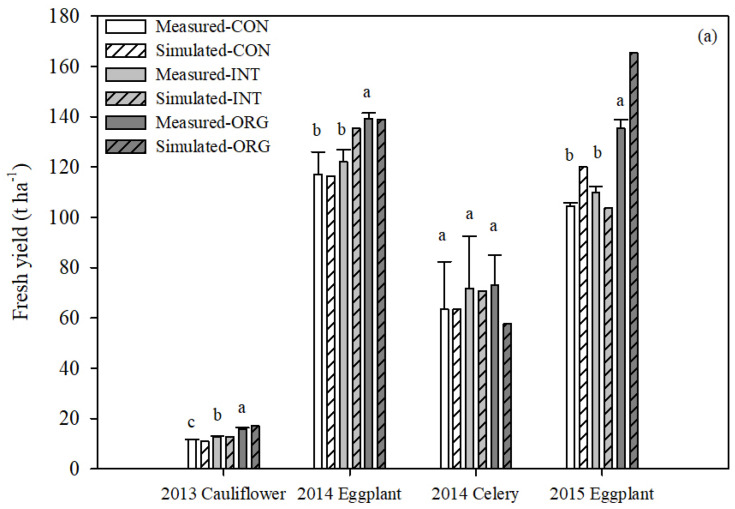

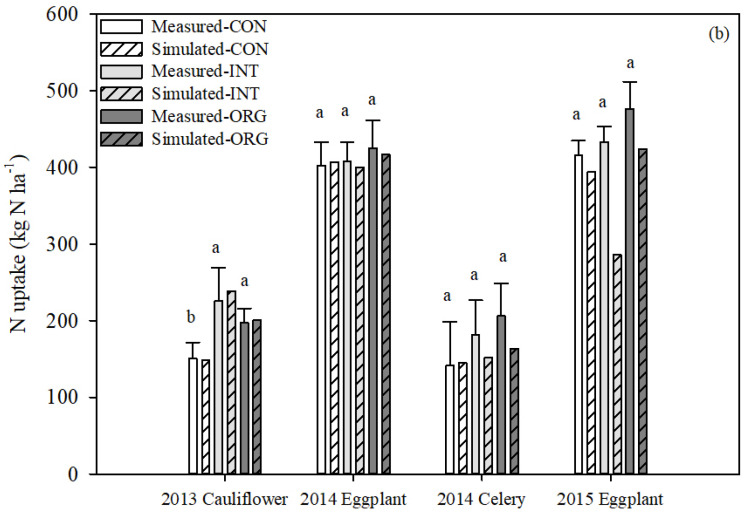
The simulated and measured vegetable yield (**a**) and N uptake (**b**) under three production systems from 2013 to 2015. (a–c represent significant differences at the *p* < 0.05 level).

Figure 3 shows the simulated and measured vegetable yields and crop N uptake under three production systems. The measured yield and crop N uptake were different for the different vegetable types, growth periods, and climate conditions. The average yield of eggplant (130.2 t ha^−1^ and 129.7 t ha^−1^) and N uptake (408.2 kg N ha^−1^ and 368.3 kg N ha^−1^) in 2014SS and 2015SS were much higher than that of cauliflower in 2013AW (average yield was 63.9 t ha^−1^, average N uptake was 153.4 kg N ha^−1^) and celery in 2014AW (average yield was 13.7 t ha^−1^, average N uptake is 196.1 kg N ha^−1^). In the AW season, the yield of celery in 2014 was 4.7 times that of cauliflower in 2013, but the N uptake of cauliflower in 2013 was 1.3 times that of celery in 2014. In the same SS season, compared with 2014SS, when the average fertilizer application rate in 2015SS decreased by 37.5%, the yield and N uptake of CON and INT systems did not decrease much, while the yield and N uptake of ORG increased by 19.0% and 1.6%, respectively. Furthermore, it is obvious that the vegetable N uptake and yield of ORG were higher than those of CON and INT. The order of two-year average vegetable yield was ORG > INT > CON. Compared with CON, the yield of ORG increased by 15.0–35.0%, and the N uptake increased by 5.6–45.5%.

For the calibrated system (CON), the *NRMSE*, *NSE*, and *d* values of the vegetable yields were 10.40%, 0.96, and 0.99, respectively. The *NRMSE*, *NSE*, and *d* values of the vegetable N uptake were 15.29%, 0.92, and 0.98, respectively. For the validation systems (INT and ORG), the *NRMSE*, *NSE*, and *d* values of vegetable yields combined for the three production systems were 4.84%, 0.94, and 1.00, respectively. The *NRMSE*, *NSE*, and *d* values of vegetable N uptake were 18.38%, 0.75, and 0.93, respectively (Table 5). Overall, the simulated vegetable yields and N uptake of each vegetable were consistent with the observed values for both the calibration and validation systems, indicating a satisfactory agreement between the model predictions and actual observations.

### 3.2. Dynamics of Soil Water Drainage and Nitrate Leaching

**Figure 4:**
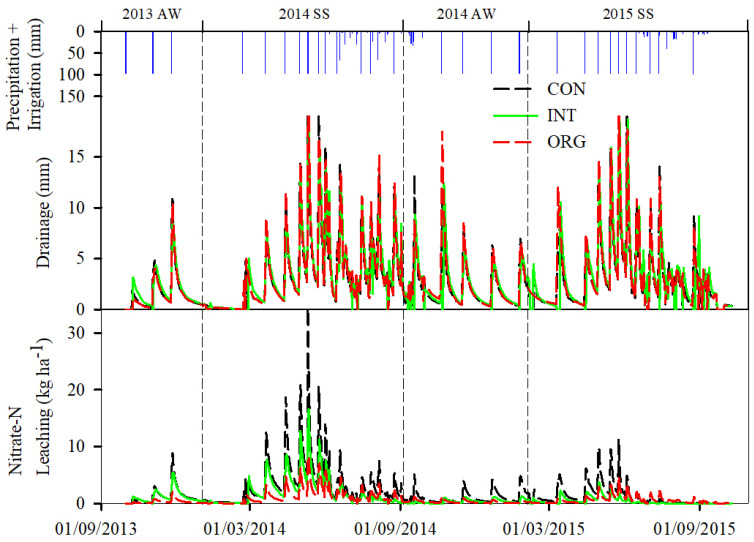
Dynamics of simulated soil water drainage and nitrate leaching under three production systems from 2013 to 2015.

Figure 4 illustrates the soil water drainage and NO_3_^−^ leaching dynamics, as simulated by the WHCNS_veg model across various soil depths within three distinct production systems. Variances in irrigation and fertilization practices throughout each season contributed to divergent patterns in soil water drainage and NO_3_^−^ leaching. Among the four vegetable seasons, due to the large amount of water input in the SS vegetable (1078 + 309 mm in 2014; 980 + 198 mm in 2015), the water drainage and NO_3_^−^ leaching exhibited significant magnitude. The average drainage in 2014SS was 881.3 mm, while the average amount of NO_3_^−^ leaching was 571.0 kg N ha^−1^. The average drainage in 2015SS was 742.8 mm, while the average NO_3_^−^ leaching rate was 171.1 kg N ha^−1^, which was obviously greater than the 2013AW, where drainage was 83.9 mm and NO_3_^−^ leaching was 148.8 kg N ha^−1^ and 2014AW, where drainage was 249.4 mm and NO_3_^−^ leaching was 171.1 kg N ha^−1^.

Soil water drainage and NO_3_^−^ leaching mainly occurred after irrigation events, especially during frequent irrigations during May–June. The irrigation amount during this period in 2014 and 2015 reached 490 mm and 588 mm, respectively, accounting for 50% of the total irrigation amount during the entire growth period, leading to increased drainage of soil water and leaching of nitrate. During the months of May and June, water drainage and nitrate leaching comprised approximately 46.2% to 51.4% and 51.0% to 56.6%, respectively, of the total drainage and leaching for the entire season. The peaks of nitrate leaching for the three production systems occurred on May 12 in 2014SS, with peak leaching of 34.4, 16.6, and 8.4 kg N ha^−1^ for CON, INT, and ORG, respectively. The difference in water drainage for different production systems was not obvious because the same irrigation amounts were applied in each system. The order of nitrate leaching under three production systems in 2014 and 2015 was CON > INT > ORG. The nitrate leaching in the ORG system was 52.3–77.8% less than in the CON system.

### 3.3. Water Balance and WUE under Three Production Systems

Table 6 presents the simulated water balance and Water Use Efficiency (WUE) for the soil profile spanning 0–100 cm, as determined by the WHCNS-Veg model. The greenhouse typically has an open roof in summer (from 27 May to 30 September in 2014 and from 1 June to 30 September in 2015) due to very high temperatures in the greenhouse. Irrigation was the main source of water during the winter (closed roof period), while irrigation and rainfall were the main sources of water during the summer (open roof period). However, irrigation was the primary source of water during the summer. Simulated water outputs included soil evaporation, crop transpiration, and water drainage. Simulated soil evaporation under the three production systems was relatively low (Table 6), ranging from 10.5 mm to 12.1 mm in the AW season and from 41.7 mm to 45.4 mm in the SS season, accounting for about 2.8–5.3% of the total water balance. However, crop transpiration was one of the main water losses and accounted for 24.9–32.1% of the total water balance. Because of the overuse of water and fertilizer N input, there was no obvious difference in vegetable yield under three production systems. Since there was no water and nutrient stress during the vegetable growth period, soil evaporation and crop transpiration of the same vegetable under three production systems were not obviously different. Evapotranspiration was much higher for the SS eggplant crops compared to the AW cauliflower and celery crops. For all production systems, water drainage accounted for 62.4–70.4% of the total water output. Therefore, water drainage was the main mechanism of water loss in these fields. Due to the large amount of water input in 2014SS and 2015SS (1087.7 + 309 mm in 2014 and 1178.4 + 198 mm in 2015), the amounts of water drainage in these two seasons were also large. The water balance of the four crop seasons was all positive, indicating that the traditional flood irrigation exceeded crop demand, which was the main reason for the large water drainage.

The WUEs of different years were different (Table 6). The WUE of the 2014AW season (56.4 to 69.2 kg m^−3^) was the highest, while the WUE of the 2013AW season (13.3 to 20.8 kg m^−3^) was the lowest, which was mainly related to the different vegetable yields. The order of WUE under three production systems in both years was ORG > INT > ORG. Compared with CON, the WUE of ORG was 19.4–37.8% higher.

### 3.4. N Fates and NUE under Three Production Systems

Table 7 illustrates the simulated outcomes of N fates and NUE within the upper 100 cm soil profile, as predicted by the WHCNS-Veg model. The main source of soil N was fertilization and net mineralization. The nitrate concentration in the irrigation water within this region ranged from 5.49 to 38.12 mg N L^−1^. Thus, the amount of nitrate brought into each system by irrigation water for the AW season ranged from 21.4 to 27.4 kg N ha^−1^, while it varied between 70.8 and 74.4 kg N ha^−1^ during the SS season, which accounted for about 5% of the total N input. The net seasonal mineralization under the three production systems was positive, which might be related to the high temperature, humidity, and strong soil microbial activity in the greenhouse vegetable field. The net mineralization of ORG was 1.6–19.5 times that of CON, and the order of net mineralization in both years was ORG > INT > CON. 

The simulated soil N output includes vegetable uptake, nitrate leaching, and gaseous loss. Vegetable N uptake was the main source of N loss from the systems (Table 7). The N uptake of cauliflower in the AW season was 145.0–238.3 kg N ha^−1^, and it was 286.0–423.9 kg N ha^−1^ for eggplant in the SS season. The N uptake in the SS season (eggplant) was much higher than that in the AW season. The N uptake component accounted for 27.3–52.2%, 39.2–69.8%, and 53.3–81.39% in the CON, INT, and ORG systems, respectively. The order of the two-year average N uptake under the three production systems was ORG > INT > CON. The N uptake of the ORG system was 5.6–45.5% greater than in the CON system. Among the three production systems, the N uptake of the ORG was the highest, while the CON was the lowest.

Nitrate leaching was the primary pathway of soil N loss. The leaching amount of the AW season ranged from 27.5 to 123.9 kg N ha^−1^, and the leaching amount of the SS season ranged from 88.2 to 884.2 kg N ha^−1^ (Table 7). The leaching amount of the SS season was much higher than that of AW. Under three production systems, the amount of nitrate leaching for ORG was lower than that of INT and CON. The CON had the largest nitrate leaching, accounting for 24.8–59.4% of the total N output. The amount of nitrate leaching under INT and ORG accounted for 21.5–50.4% and 11.5–40.0% of the total N output, respectively. The amount of nitrate leaching under ORG was 52.3–77.8% lower than that of CON. The nitrate leaching under three production systems followed the order: CON > INT > ORG. The nitrate leaching accounted for 11.5–59.4% of the total N output.

The gaseous N loss was another primary pathway of soil N loss. The gaseous N loss in a single season reached 12.2–187.6 kg N ha^−1^, accounting for 6.0–21.1% of the N losses (Table 7). The gaseous N loss of CON, INT, and ORG was 9.7–21.1%, 8.6–12.6%, and 6.0–9.2% of the total N loss, respectively. The gaseous N loss in different seasons was also different. In general, the gaseous N loss during the SS season was higher than that of the AW season. The average gaseous N loss in the 2014AW season was the lowest (34.2 kg N ha^−1^), and it was the highest (114.8 kg N ha^−1^) in the 2014SS season. The average gaseous N loss per season showed the order of CON (98.5 kg N ha^−1^) > INT (53.0 kg N ha^−1^) > ORG (34.2 kg N ha^−1^), in which the losses in the ORG system was 22.1–80.1% less than that of CON. 

Among the four vegetable crops, the NUE of the 2013AW was the lowest, ranging from 32.5 to 70.0 kg kg^−1^, and the NUE of the AW crop in 2014 was the highest, ranging from 192.0 to 306.8 kg kg^−1^. The NUE of the three production systems showed the order of ORG > INT > CON. The NUE of the ORG production system was 47.8–115.3% higher for the AW season and 68.6–127.0% higher for the SS season compared with the CON treatment. The NUE also varied from year to year. Among them, the NUE of the AW season in 2014 was the highest, while the NUE of the AW season in 2013 was the lowest. 

Among the four-season vegetables, the NUE of the 2013AW season was the lowest (32.5–70.0 kg kg^−1^), and the NUE of the 2014AW season was the highest (192.0–306.8 kg kg^−1^). The NUE under three production systems showed the order of ORG > INT > CON. Compared with the CON, the NUE of ORG was 47.8–115.3% higher in the AW season and 68.6–127.0% in the SS season.

## 4. Discussion

### 4.1. The Effects of Production Systems on Vegetable Yield and N Uptake

Substituting organic fertilizers for chemical fertilizers could increase the crop yield. Wei et al. (2016) summarized 32 long-term experimental datasets in China by meta-analysis and showed that compared with the traditional practice, substituting part chemical fertilizers with organic fertilizers could increase yield by 29% [43]. Zhang et al. (2018) found that only applying chemical fertilizers or the combined application of organic and inorganic fertilizers could increase yields by 11–13% compared with the traditional farmers’ practices in the Taihu Lake area [44]. Li et al. (2018) demonstrated a yield increase of 9.9–17.4% by employing both organic and inorganic fertilizers, surpassing the yield obtained solely from chemical fertilizer application [45]. Long-term use of organic fertilizers has enhanced soil quality, including improvements in water retention capacity, conductivity, and overall structure, increased soil organic carbon and other nutrients content, reduced N loss, and improved crop yield [28,29,30]. In this study, the order of vegetable yield under three production systems was ORG > INT > CON. Compared with the CON system, the ORG system yield increased by 15.0–35.0%. The CON system received similar or higher nitrogen rates than the ORG system, and the nitrogen in the CON system is mainly in the form of chemical fertilizer, which is easily lost by leaching and gas emissions (Table 6). The available soil mineral N in the CON system is less than that in the ORG system, which led to the yield of the ORG system being higher than that of the CON system. The higher yield observed in organic farming systems contrasts with findings from certain earlier studies [14,46]. There are two reasons for that: (1) Usually, vegetables like to have plenty of water and fertilizer. The organic fertilizer input for organic farming systems in this study was much higher than those in previous studies, which can provide enough nutrients to meet the requirement during the whole period of vegetable growth. In contrast, the relatively low input of organic fertilizers in previous studies could not supply sufficient nutrients for crop growth and led to low yields in organic farming systems. (2) Long-term application of organic manure in the organic farming system could improve soil structure and increase soil water and nutrient retention capacity [47], which provides a good condition for vegetable growth.

Substituting organic fertilizers for chemical fertilizers could also increase the crop N uptake. Xia et al. (2017) found that substituting chemical fertilizers with organic fertilizers could increase N uptake by 8.8% through a meta-analysis of 141 study areas [48]. Zhang et al. (2019) reported that substituting compound fertilizers with manure and biochar increased the N uptake of vegetables by 54.1% in a greenhouse in Daxing of Beijing [49]. In a meta-analysis of 502 sets of observational data in China’s vegetable production system, Liu et al. (2021) discovered that the combined use of organic and chemical fertilizers led to a 15.9% decrease in nitrogen uptake, in contrast to sole chemical fertilizer application [26]. Zhou et al. (2022) conducted a 15-year greenhouse tomato cultivation experiment in Shouguang, Shandong Province, and found that combined optimal fertilization with straw addition could reduce N leaching by 4–86% without reducing tomato yield and N uptake [50]. In this study, ORG exhibited a 5.6% to 45.5% increase in N uptake compared to CON. These findings are consistent with prior research.

### 4.2. The Effects of Production Systems on Nitrate Leaching

Nitrate leaching stands out as a primary route through which nitrogen is lost in greenhouse vegetable fields and is closely associated with irrigation methods and amounts [10], fertilization management [3], and planting catch crops [51]. In this research, flood irrigation was employed, with each application delivering approximately 100 mm of water, and the total irrigation amounts of the four vegetable crops were 294 mm, 1078 mm, 392 mm, and 980 mm, respectively. Excessive irrigation resulted in the large nitrate leaching. The CON system had the largest nitrate leaching, 117.9–884.2 kg N ha^−1^, accounting for 34.9–59.8% of the total N output. The amount of nitrate leaching under the INT and ORG systems was 50.5–515.6 kg N ha^−1^ and 27.5–313.2 kg N ha^−1^, respectively, accounting for 21.5–50.4% and 11.5–40.0% of the total N output, respectively. The nitrate leaching amount in this study was much higher than the average nitrate leaching amount in Chinese greenhouse vegetable systems, which is 98.0 kg N ha^−1^ over the entire crop growing season [3]. Therefore, it is essential to further optimize the levels of irrigation and fertilization within the study area.

Fertilization also has a significant effect on the nitrate leaching in the greenhouse vegetable fields. Wang et al. (2019) showed that reducing the N application rate by 20% and 50% during the entire vegetable growing season could reduce nitrate leaching by 18.3% and 43.0%, respectively [52]. Zhou et al. (2022) reported that combined optimal fertilization with straw addition could reduce nitrate leaching by 4–86% in a 15-year greenhouse tomato experiment in Shouguang County, Shandong Province [50]. In this study, the amounts of nitrate leaching under the CON, INT, and ORG systems accounted for 34.8–59.8%, 21.5–50.4, and 11.5–40.0% of the total N input, respectively. The average amount of nitrate leaching per season of the CON system (353.5 kg N ha^−1^) was much higher than those of the INT (189.9 kg N ha^−1^) and ORG (126.6 kg N ha^−1^) systems. The main reason was that the CON system had the highest total fertilizer input, and the ratio of chemical fertilizers accounted for 70%. In addition, the leaching nitrate amount in this study was much higher than in the previous studies [3,10], which was closely related to the large fertilization amount in this study.

Many studies have shown that substituting organic fertilizers for chemical fertilizers in vegetable production systems could promote N fixation and reduce nitrate leaching [3,29,53]. Xia et al. (2017) found that in 141 study areas, substituting chemical fertilizers with organic fertilizers reduced nitrate leaching by 28.9% through a meta-analysis [48]. Wei et al. (2021) found that substituting organic fertilizers for chemical fertilizers reduced nitrate leaching by more than 30% without reducing vegetable yield [27]. The CON system in this study applied chemical fertilizers with the highest nitrate leaching amount, while the ORG system only applied organic fertilizers and gave the lowest nitrate leaching amount. Compared with the CON, the ORG system reduced the nitrate leaching amount by 52.3–77.8%, reducing the pollution risk to the environment, which was consistent with previous reports [3,44].

### 4.3. The Effects of Production Systems on Gaseous N Loss

Another primary pathway of nitrogen (N) loss in greenhouse vegetable fields is through the volatilization of gaseous nitrogen [54,55,56]. The gaseous N loss for vegetable production systems is related to fertilization management [57,58], vegetable species, residue treatment [59,60], climatic and soil conditions [61,62]. 

Reducing N application rates can reduce the gaseous N loss. He et al. (2009) showed that reducing N application rates by 69% and 76% reduced gaseous N loss by 51% and 27%, respectively, compared with traditional N management [63]. Huang et al. (2017) showed that reducing the fertilizer amount by 50% of the fertilization rate based on traditional treatment could reduce N_2_O emissions by 18–37% [58]. During the growing season of 2015SS, the fertilizer application rates for the CON and INT systems were reduced by 44.9% and 38.4%, respectively, compared to 2014SS. Additionally, the gaseous N loss in 2015SS decreased by 60.9% and 66.3%, respectively, compared to the previous year.

Replacing chemical fertilizers with organic ones can notably decrease the emission of gaseous nitrogen in vegetable cultivation systems [44]. Xia et al. (2017) found that substituting chemical fertilizers with organic fertilizers reduced NH_3_ volatilization by 26.8% [48]. In a meta-analysis of 502 sets of observational data in China’s vegetable production system, Liu et al. (2021) found that when the substitution ratio was >70%, N_2_O emissions were reduced by 14.3%, and there was no significant change in NH_3_ volatilization, which might be affected by factors such as the organic fertilizer type and soil pH [26]. Zhang et al. (2019) showed that substituting compound fertilizer with manure and biochar reduced the gaseous N loss by 7.4% in a solar greenhouse in suburban Beijing [49]. In this study, the fertilizer type applied in the CON was mainly urea, and the gaseous N loss was the largest, while the ORG only applied organic fertilizer. Compared to the CON, the ORG reduced the gaseous N loss by 63.2%. Thus, the application of organic fertilizer obviously reduced the gaseous N loss, which was consistent with the previous research results.

## 5. Conclusions

The WHCNS-Veg model underwent calibration and validation utilizing a field dataset consisting of four seasons of vegetables grown from 2013 to 2015 under three production systems. The results suggested that the WHCNS-Veg model effectively replicated soil moisture, nitrate levels, vegetable yield, and nitrogen uptake, demonstrating a strong agreement with the observed data. Therefore, the WHCSN-Veg could be used to quantitatively analyze the water balance and N fates of the greenhouse in the study area.

The simulation results revealed that nitrate leaching and gaseous nitrogen loss emerged as the primary routes of nitrogen loss within the greenhouse vegetable production system. The nitrate leaching rate of the four vegetables accounted for 11.5–59.4% of the total N output. The gaseous N loss in a single season reached 12.2–187.6 kg N ha^−1^, accounting for 6.0–21.1% of the N output. From 2013 to 2015, the orders of crop yield, N uptake, WUE, and NUE were generally followed: ORG > INT > CON, while the order of nitrate leaching and gaseous N loss was CON > INT > ORG. The ORG system increased vegetable yield (increased by 15.0–35.0%), N uptake (increased by 5.6–45.5%), WUE (increased by 19.4–37.8%), and NUE (increased by 47.8–127.0%), while reducing nitrate leaching (52.3–77.8%) and gaseous N loss (71.7–90.5%). Therefore, the ORG is a preferable production system for greenhouse vegetables in this region.

## Figures and Tables

**Table 1:** Planting information and fertilizer N input under three production systems from 2013 to 2015 (kg N ha^−1^).

Season	Vegetable	Transplanting Date	Harvest Date	Treatment	Organic Fertilizer	Chemical Fertilizer	Total Amount
2013AW	Cauliflower			CON	235	350	585
		1 October	3 January 2014	INT	399	175	574
				ORG	798	/	798
2014SS	Eggplant			CON	227	992	1219
		20 February	6 September	INT	459	496	954
				ORG	917	/	917
2014AW	Celery			CON	149	350	499
		19 October	2 February 2015	INT	254	175	429
				ORG	507	/	507
2015SS	Eggplant			CON	146	525	671
		11 March	18 September	INT	325	263	588
				ORG	651	/	651

Note: CON refers to conventional farming, INT to integrated farming, and ORG to organic farming systems. AW denotes the autumn–winter season, while SS indicates the spring–summer season.

**Table 2:** Soil physical and hydraulic properties for the 0–100 cm soil profile.

Soil Layer (cm)	Treatment	BD	Particle Fraction (%)			*θ_r_*	*θ_s_*	*θ_fc_*	*θ_wp_*	*K_s_*
			Sand	Silt	Clay					
	CON	1.53				0.07	0.40	0.28	0.14	21.3
0–20	INT	1.24	59.7	36.9	3.4	0.08	0.42	0.30	0.15	23.7
	ORG	1.13				0.09	0.43	0.32	0.16	24.8
20–40		1.49	10.1	75.9	14.0	0.07	0.34	0.27	0.10	16.1
40–60		1.44	10.1	77.9	12.0	0.07	0.36	0.26	0.11	18.8
60–80		1.36	14.1	71.9	14.0	0.07	0.36	0.25	0.11	24.6
80–100		1.36	6.1	85.9	8.0	0.07	0.40	0.25	0.11	28.1

Note: BD is bulk density (g cm^−3^); *θ_r_* is the residual soil water content (cm^3^ cm^−3^); *θ_s_* is the saturated soil water content (cm^3^ cm^−3^); *θ_fc_* is the field capacity (cm^3^ cm^−3^); *θ_wp_* is the wilting point (cm^3^ cm^−3^); *K_s_* is the saturated hydraulic conductivity (cm d^−1^).

**Table 3:** Soil basic chemical properties in surface soil (0–20 cm) before September 2013.

Treatment	pH	Soil Organic Matter (g kg^−1^)	Total N (g kg^−1^)	Nitrate-N (mg kg^−1^)	Available P (mg kg^−1^)	Available K (mg kg^−1^)
CON	7.73	22.4	1.8	9.96	247.2	556.0
INT	7.57	27.9	2.0	24.9	319.1	560.0
ORG	7.45	46.6	2.9	9.8	552. 5	533.7

**Table 4:** The input parameters of the WHCNS-Veg model.

Groups	Parameters	Description	Vegetable		
			Cauliflower	Celery	Eggplant
	*T_base_*	Base temperature (°C)	5	4	15
	*T_sum_*	Accumulated available temperature (°C)	800	850	1500
	*K_ini_*	Crop coefficient in the initial stage (-)	0.7	0.8	0.9
Crop of	*K_mid_*	Crop coefficient in the middle stage (-)	1.3	1.4	1.5
parameters	*K_end_*	Crop coefficient at the end stage (-)	1	1.1	1.2
	*R_max_*	Maximum root depth (cm)	20	20	50
	*α_DM_*	Dry matter accumulation empirical constant (t ha^−1^)	1	1	1
	*Nmin*	Minimum N concentration of plant (%)	3.3	1.5	3
	*α_N_*	Empirical parameters of critical function (-)	17	15	5
	*V_n_*	Maximum nitrification rate (mg L^−1^ d^−1^)	30		
	*K_n_*	Half saturation constant (mg L^−1^)	100		
Parameters of N	*K_d_*	An empirical proportionality factor (mg mg^−1^)	1.5		
transformation	*A_d_*	Empirical coefficient (-)	0.3		
parameters	*K_v_*	First-order kinetic constant of volatilization (d^−1^)	0.1		
	*R_nit_*	The ratio of N_2_O produced by the nitrification process (-)	0.01		
	*R_den_*	The ratio of N_2_O produced by the denitrification process (-)	0.5		

**Table 5:** Calibration and validation results between simulated and measured soil water content, nitrate concentration, and plant variables.

	Soil Layers cm	Calibration (CON)			Validation (INT and ORG)		
		*NRMSE* (%)	*NSE*	*d*	*NRMSE* (%)	*NSE*	*d*
Soil water content	0–20	5.91	0.56	0.89	6.47	0.47	0.88
	20–40	5.65	0.60	0.88	4.19	0.62	0.90
	40–60	3.65	0.78	0.94	4.83	0.62	0.90
	60–80	6.36	0.59	0.89	5.27	0.79	0.95
	80–100	9.17	0.50	0.89	8.34	0.55	0.91
Soil nitrate concentration	0–20	27.41	0.90	0.98	25.04	0.91	0.98
	20–40	26.30	0.91	0.98	29.78	0.89	0.98
	40–60	23.30	0.92	0.98	28.19	0.86	0.97
	60–80	24.18	0.91	0.98	29.09	0.84	0.96
	80–100	19.88	0.93	0.98	27.48	0.82	0.96
Fresh yield		10.40	0.96	0.99	4.84	0.99	1.00
Vegetable N uptake		15.29	0.92	0.98	18.38	0.75	0.93

**Table 6:** Water balance under three production systems simulated by WHCNS-Veg from 2013 to 2015.

Year	Vegetable	Treatment	I (mm)	P (mm)	E (mm)	T (mm)	ET (mm)	D (mm)	Wbal (mm)	Y (t ha^−1^)	WUE (kg m^−3^)
2013AW		CON	294	0	12.1	70.3	82.4	145.4	66.1	11.0	13.3
	Cauliflower	INT	294	0	12.1	70.3	82.4	164.1	47.5	12.8	15.5
		ORG	294	0	12.1	70.3	82.4	137.0	74.6	17.2	20.8
2014SS		CON	1078	309	45.4	400.3	445.7	880.3	61.8	116.4	26.1
	Eggplant	INT	1078	309	45.4	400.2	445.6	881.1	61.1	135.3	30.4
		ORG	1078	309	45.4	400.3	445.7	882.4	59.7	139.0	31.2
2014AW		CON	392	0	10.5	91.8	102.3	243.1	46.6	63.5	62.1
	Celery	INT	392	0	10.5	91.8	102.3	238.1	51.6	70.5	69.2
		ORG	392	0	10.5	91.8	102.3	267.1	22.6	57.7	56.4
2015 SS		CON	980	198	41.7	367.0	408.7	735.8	33.9	120.0	29.4
	Eggplant	INT	980	198	41.7	366.7	408.4	736.7	33.3	103.6	25.4
		ORG	980	198	41.7	366.9	408.6	755.8	14.0	165.4	40.5

Note: I, irrigation; P, precipitation; E, soil evaporation; T, vegetable transpiration; ET, actual evapotranspiration; D, drainage; Wbal, water balance, Wbal = I + P − E − T − D; Y, fresh yield. WUE, water use efficiency = Y/ET.

**Table 7:** N fate and NUE under three production systems simulated by WHCNS-Veg from 2013 to 2015.

Season	Vegetable	Treatment	N Input (kg N ha^−1^)			N Output (kg N ha^−1^)			NUE (kg kg^−1^)
			F	I	Nmin	Nup	Nlea	Ngas	
2013AW		CON	350.0	27.4	253.6	148.7	117.9	71.6	32.5
	Cauliflower	INT	175.0	27.4	385.5	238.3	105.7	42.3	33.1
		ORG	0.0	27.4	406.9	201.3	28.2	15.8	70.0
2014SS		CON	991.7	74.4	33.8	406.9	884.2	187.6	78.2
	Eggplant	INT	495.8	74.4	141.8	400.3	515.6	105.2	132.3
		ORG	0.0	74.4	437.6	417.4	313.2	51.5	177.5
2014AW		CON	350.0	21.4	15.1	145.0	123.9	61.3	192.0
	Celery	INT	175.0	21.4	49.6	151.6	50.0	29.1	306.8
		ORG	0.0	21.4	202.5	163.6	27.5	12.2	283.8
2015SS		CON	525.0	70.8	26.1	394.9	287.8	73.3	158.7
	Eggplant	INT	262.5	70.8	101.2	286.0	88.2	35.3	253.0
		ORG	0.0	70.8	509.7	423.9	137.4	57.1	267.5

Note: F, fertilizer; I, Irrigation; Nmin, mineralization N; Nup, uptake N; Nlea, N leaching; Ngas, gaseous N loss; NUE, N use efficiency.

## Data Availability

Data are contained within the article.
